# Red blood cell distribution width-to-albumin ratio and in-hospital major adverse cardiovascular events in patients with acute myocardial infarction complicated by cardiogenic shock: a two-center retrospective cohort study

**DOI:** 10.3389/fcvm.2026.1866356

**Published:** 2026-08-03

**Authors:** Jia Liu, Tong Wang, Yijie Huang, Xudong Li, Bing Han, Pengsheng Chen

**Affiliations:** 1 Xuzhou Central Hospital, Southeast University, Xuzhou, Jiangsu, China; 2 The First People’s Hospital of Yancheng, Yancheng, China; 3 Baoji People’s Hospital, Baoji, China

**Keywords:** acute myocardial infarction with cardiogenic shock (AMI-CS), albumin, major adverse cardiovascular events (MACE), red blood cell distribution width (RDW), red blood cell distribution width-to-albumin ratio (RAR)

## Abstract

**Objective:**

To investigate the relationship between red blood cell distribution width-to-albumin ratio (RAR) and major adverse cardiovascular events (MACE) in patients with acute myocardial infarction complicated by cardiogenic shock (AMI-CS) during hospitalization.

**Methods:**

This retrospective cohort study enrolled patients from Xuzhou Central Hospital and the First People's Hospital of Yancheng between January 2019 and December 2024. Patients were stratified into four quartiles according to admission RAR. The relationship between RAR and in-hospital MACE (all-cause death, recurrent non-fatal myocardial infarction, unplanned revascularization, non-fatal stroke) was assessed using multivariate logistic regression, Kaplan–Meier curves, and receiver operating characteristic (ROC) analysis.

**Results:**

A total of 3,316 AMI patients were screened and 264 AMI-CS patients were enrolled in this study. Mortality rates across RAR quartiles (lowest to highest) were 33.3%, 37.9%, 54.5%, and 66.7%, and MACE rates were 54.5%, 53.0%, 62.1%, and 78.8% (*P* < 0.001). After adjustment for confounders, elevated RAR was an independent predictor of in-hospital MACE (odds ratio [OR] = 2.063, 95% confidence interval [CI]: 1.374–3.009, *P* < 0.001) and mortality (OR = 2.033, 95% CI: 1.341–3.084, *P* = 0.001). The highest RAR quartile was associated with a 4.008-fold higher risk of MACE (95% CI: 1.504–10.680, *P* = 0.006) and a 3.354-fold higher risk of mortality (95% CI: 1.414–7.958, *P* = 0.006) compared with the lowest quartile. Kaplan–Meier curves showed that higher RAR quartiles were associated with higher rates of MACE and mortality (*P* < 0.001). RAR exhibited better predictive performance than RDW or albumin alone, with an area under the curve (AUC) of 0.734 (cutoff = 4.335) for MACE and 0.716 (cutoff = 4.400) for mortality.

**Conclusion:**

RAR showed higher discriminatory performance than RDW or albumin alone in AMI-CS patients and may be considered a candidate prognostic marker requiring further validation.

**Clinical Trial Registration:** https://www.clinicaltrials.gov, identifier NCT07507110.

## Introduction

Coronary atherosclerotic heart disease remains the leading cause of global mortality (1). In China, the incidence and mortality of acute myocardial infarction (AMI) have continued to rise, imposing substantial clinical and economic burdens on the healthcare system (2). Despite remarkable progress in reperfusion strategies and guideline-directed medical management, the in-hospital mortality rate for AMI persists above 4% (3, 4). This high mortality is mainly driven by severe acute complications, including heart failure, malignant arrhythmias, and cardiogenic shock—among which cardiogenic shock represents the most life-threatening condition (5). Approximately 5% of AMI patients develop cardiogenic shock during hospitalization, and these patients face a sharp increase in 60-day all-cause mortality with persistently poor long-term outcomes (3, 6).

The identification of reliable prognostic biomarkers for AMI complicated by cardiogenic shock (AMI-CS) is critical for early risk stratification and guiding individualized treatment strategies. Although conventional biomarkers such as NT-proBNP, troponin, and C-reactive protein are widely used in clinical practice, their application is constrained by relatively high costs, delayed laboratory turnaround times, and suboptimal predictive accuracy for adverse outcomes. Furthermore, existing biomarkers have not been adequately validated within established AMI risk stratification frameworks to accurately predict prognosis in the specific high-risk cohort of AMI-CS patients. Consequently, there is a pressing clinical need for a readily available, cost-effective, and robust prognostic indicator to enhance risk assessment and therapeutic decision-making for this critically ill population.

Red blood cell distribution width (RDW) is a routine hematological parameter that quantifies variability in erythrocyte volume (7). Mounting evidence has demonstrated that RDW is closely associated with adverse cardiovascular outcomes, partly reflecting systemic inflammation and oxidative stress (8). Previous studies have reported that elevated RDW is an independent predictor of six-month mortality in AMI and long-term all-cause mortality in patients with STEMI undergoing revascularization (9). Accumulating evidence further suggests that the combination of RDW with other biomarkers may yield superior prognostic value compared with either marker alone (8, 10). Beyond RDW alone, other RDW-derived hematologic indices, such as the hemoglobin-to-red cell distribution width ratio, have also been evaluated in emergency department patients with acute coronary syndrome and have shown associations with short-term mortality and major adverse cardiovascular events (MACE), supporting the potential prognostic relevance of simple complete blood count-derived ratios in acute coronary presentations. However, current RDW-derived hematologic indices could not well reflect the prognosis of AMI-CS (11).

Albumin is a major serum protein routinely measured in clinical practice. It serves as an important indicator of nutritional status and systemic health, and exhibits anti-inflammatory and antioxidative properties (12, 13). Hypoalbuminemia has been associated with poor prognosis in various cardiovascular disorders, likely indicating sustained inflammation and malnutrition (14). Recent studies have confirmed that low serum albumin level predicts adverse outcomes in patients with AMI (15, 16).

Given that both RDW and albumin are independently associated with cardiovascular risk, their combined index, the red blood cell distribution width-to-albumin ratio (RAR), has emerged as a novel prognostic marker in AMI, but its value remains incompletely understood (8). RAR may provide a more integrated assessment of inflammation, nutritional status, and hemodynamic stress, thereby facilitating early identification of high-risk AMI patients who may benefit from intensified monitoring and intervention. However, the short-term prognostic significance of RAR in AMI-CS patients remains unclear. Accordingly, the present study aimed to investigate the association between RAR and in-hospital MACE in patients with AMI-CS.

## Materials and methods

### Study design and participants

This was a retrospective, two-center study conducted at Xuzhou Central Hospital and The First People's Hospital of Yancheng. Between January 2019 and December 2024, a total of 3,316 consecutive patients diagnosed with AMI were screened. Among them, 264 cases complicating with cardiogenic shock were included. The inclusion criteria were as follows: (1) Age 18–90 years; (2) Confirmation of AMI per current guidelines (17): elevation of myocardial injury markers (preferably troponin) ≥the 99th percentile upper reference limit, plus at least one sign of myocardial ischemia (persistent ischemic symptoms ≥30 min, ST-segment deviation on electrocardiogram); (3) Diagnosis of cardiogenic shock per consensus criteria (18): systolic blood pressure <90 mmHg, accompanied by pulmonary congestion (e.g., dyspnea) and at least one sign of end-organ hypoperfusion: altered mental status, cool clammy skin, urine output <30 mL/h, or serum lactate >2.0 mmol/L. The exclusion criteria included: (1) Coexisting cerebrovascular disease, peripheral vascular disease or embolic disease; (2) Myocardial infarction caused by coronary artery embolism or blood flow interruption due to peripheral embolus detachment or invasive diagnostic and therapeutic procedures; (3) History of surgery, trauma or infection within one month; (4) History of severe liver or kidney dysfunction, including alanine aminotransferase (ALT), aspartate aminotransferase (AST), and γ-glutamyl transpeptidase (γ-GGT) > 2.5 times upper limit of normal value; Serum creatinine >1.5 times upper limit of normal value; (5) Coexisting autoimmune diseases, tumors, etc. This study was conducted in accordance with the Declaration of Helsinki of Good Clinical Practice, and was approved by the Research Ethics Board of the Xuzhou Central Hospital (XZXY-LJ-20210715-056).

### Data collection

Baseline data were extracted from electronic medical records within 24 h of admission, including demographics (age, gender), medical history (smoking, alcohol use, prior medications), vital signs (systolic/diastolic blood pressure, heart rate), laboratory results (hemoglobin, white blood cell count, platelet count, lipid profile, serum creatinine, B-type natriuretic peptide, C-reactive protein, cardiac troponin I), and echocardiographic parameters.

### Groups and outcomes

RDW was measured by Sysmex XN10 hematology analyzer, with a reference range of 10.1%–14.8%. Serum albumin levels were measured using standard laboratory techniques. The RAR was defined as the ratio of RDW value to albumin level (%/g/dL). Patients were then stratified into four quartiles groups based on their RAR values. The primary endpoint was the occurrence of major adverse cardiovascular events (MACE) during hospitalization, encompassing all-cause death, recurrent non-fatal myocardial infarction, unplanned revascularization, and non-fatal stroke. Secondary endpoints included the individual components of MACE. Deaths were classified as cardiovascular, non-cardiovascular, or of unknown cause. Non-cardiovascular death required clear documentation of a non-cardiac etiology, and multiorgan failure secondary to cardiovascular disease was adjudicated as cardiovascular death (19). Unplanned revascularization was defined as the unplanned PCI or CABG surgery performed for recurrent ischemic symptoms associated with objective features of ischemia, e.g., ECG changes or positive functional testing (20). Recurrent non-fatal myocardial infarction was defined as follows: 1) New ST elevation of ≥1 mm in at least 2 contiguous leads and recurrent cardiac ischemic symptoms ≥20 min (21); 2) Index AMI with unresolved myocardial necrosis biomarkers: ≥50% increase in troponin (decreasing trend) plus either recurrent rest angina ≥20 min, new ST-segment elevation ≥1 mm in ≥2 contiguous leads, new pathological Q waves, or new left bundle branch block (21); 3) Reinfarction after normalized myocardial necrosis biomarkers (excluding PCI within 24 h): troponin re-elevation consistent with acute MI plus recurrent rest angina ≥20 min or new ischemic ECG changes (21). Stroke was defined as a new focal vascular neurologic deficit lasting >24 h, confirmed by computed tomography (CT) or magnetic resonance imaging (MRI) when available (21). Events were identified from standardized records and the endpoints were adjudicated by two independent researchers who were blinded to RAR values. If they had disagreements, a third independent researcher would make the final decision.

### IABP-SHOCK II risk scores

IABP-SHOCK II (intra-aortic balloon pump SHOCK II) risk scores were used to assess shock severity, with the scoring criteria as follows: 1 point for age >73 years at admission, 2 points for a history of previous stroke, 1 point for blood glucose >10.6 mmol/L, 1 point for serum creatinine >132.6 μmol/L, 2 points for blood lactate >5 mmol/L, and 2 points for post-intervention TIMI flow grade <3. Patients were stratified into low risk (total score 0–2), moderate risk (total score 3–4), and high risk (total score 5–9) (22).

### Statistical analysis

Continuous data are presented as mean ± standard deviation (SD) or median (interquartile range, IQR). Distribution normality was assessed with the Kolmogorov–Smirnov test. Between-group differences in continuous variables were compared using Student's *t*-test for normally distributed data, or the Mann–Whitney *U*-test for non-normally distributed data. Categorical variables are reported as numbers and percentages, and between-group differences were evaluated using the *χ*^2^ test or Fisher's exact test, as appropriate.

Multivariate logistic regression analysis was performed to calculate the odds ratio (OR) and 95% confidence interval (95% CI) for the association between RAR and in-hospital MACE and all-cause mortality in AMI-CS patients. Outcomes were reported across three models: Model 1 (unadjusted), Model 2 (adjusted for age, sex, and medical history), and Model 3 (additionally adjusted for age, sex, medical history, systolic/diastolic blood pressure, hemoglobin, white blood cell count, alanine aminotransferase, total cholesterol, triglycerides, serum creatinine, fasting blood glucose, cardiac troponin I, C-reactive protein, B-type natriuretic peptide), based on prior literature (8, 10).

Kaplan–Meier survival curves and the log-rank test were used to characterize survival distributions. The day of confirmed diagnosis at admission was defined as time zero, the day of MACE occurrence was recorded as the event time, and all patients were censored at hospital discharge or 30 days, whichever occurred first. Receiver operating characteristic (ROC) curve analysis was performed to evaluate the predictive accuracy of RAR for in-hospital MACE, and we compared the predictive performance of RAR with that of RDW and albumin to confirm its incremental prognostic value. The areas under the curves (AUCs) were compared using the Delong test.

For the handling of missing values, a standardized processing procedure was adopted (23). Briefly, the first step is defining the missing data mechanisms of each variable. All clinical events were completely recorded. And rare laboratory results like B-type natriuretic peptide, C-reactive protein, and echocardiographic parameters results were missing. Meanwhile, since most patients were in a bedridden state due to their critical condition, there were many missing BMI data. To avoid affecting the statistical results, we directly excluded these data in our analysis. The missing data were used multiple interpolation method and the results were tested by Sensitivity analyses.

All tests were two-sided, and a *P*-value <0.05 was considered statistically significant. All analyses were performed using SPSS version 22.0 (IBM Corp., Armonk, NY, USA) or SAS version 9.4 (SAS Institute, Cary, NC, USA).

## Results

### Baseline characteristics

**Figure 1:**
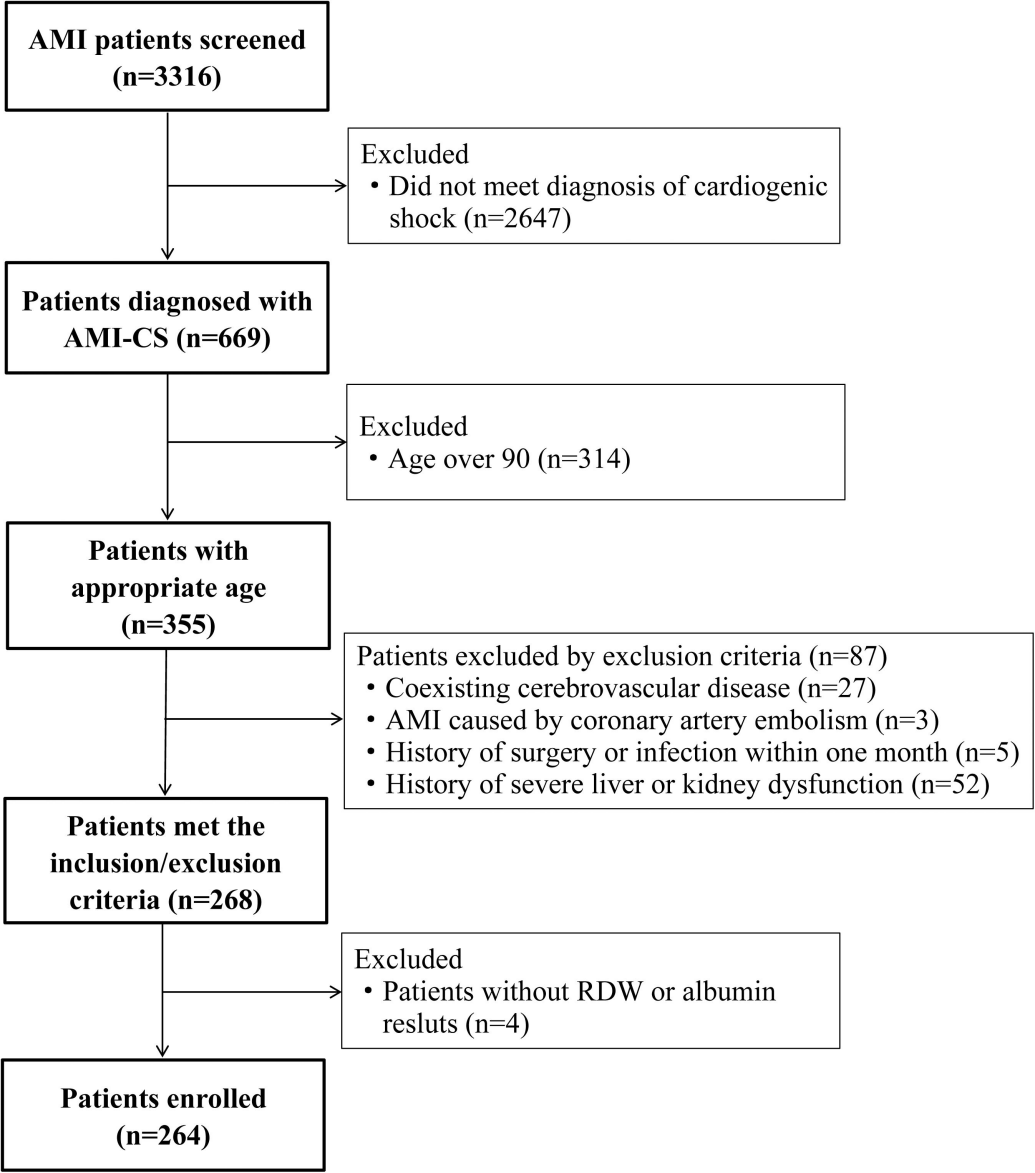
Study flow chart. AMI indicates acute myocardial infarction; AMI-CS, acute myocardial infarction complicated with cardiogenic shock; RDW, red blood cell distribution width.

A total of 3,316 patients diagnosed with AMI were screened, and 264 with cardiogenic shock were included. Study flow diagram was shown in Figure 1. Mean age was 66.33 ± 12.40 years, and 67.5% were male. Mean RAR was 4.01 ± 1.00%/g/dL; mean RDW and albumin were 13.93 ± 2.04% and 35.88 ± 5.94 g/L, respectively. Patients were divided into four RAR quartiles (≤3.31, 3.32–3.80, 3.81–4.47, >4.47%/g/dL). Baseline characteristics are presented in Table 1. Mortality rates across RAR quartiles (lowest to highest) were 33.3%, 37.9%, 54.5%, and 66.7%, and MACE rates were 54.5%, 53.0%, 62.1%, and 78.8% (*P* < 0.001). Patients in the highest RAR quartile also had significantly higher IABP-SHOCK II scores (*P* < 0.001). The lowest RAR quartile was associated with lower MACE and mortality rates, and rates increased progressively with rising RAR (*P* < 0.001).

**Table 1:** Baseline characteristics of the included participants.

Characteristics	RAR value (%/g/dL)				*P* value
	Q1 (*N* = 66)	Q2 (*N* = 66)	Q3 (*N* = 66)	Q4 (*N* = 66)	
Age (±SD)—years	64.70 ± 11.90	65.06 ± 11.334	63.73 ± 11.41	67.83 ± 14.56	0.263
Male sex—no. (%)	48 (72.7)	38 (57.6)	44 (66.7)	51 (77.3)	0.084
SBP (±SD)—mmHg	120.20 ± 25.61	111.58 ± 19.81	102.74 ± 20.76	105.15 ± 22.73	**<0**.**001**
DBP (±SD)—mmHg	74.23 ± 25.16	68.23 ± 14.36	65.58 ± 14.03	65.12 ± 15.81	**0**.**014**
Hypertension—no. (%)	36 (54.5)	26 (39.4)	28 (42.4)	32 (48.5)	0.308
Diabetes mellitus—no. (%)	21 (31.8)	23 (34.8)	18 (27.3)	19 (28.9)	0.789
Current smoker—no. (%)	13 (19.7)	13 (19.7)	16 (24.2)	21 (31.8)	0.312
RDW (±SD)—%	12.59 ± 0.67	13.19 ± 0.73	14.12 ± 1.16	15.82 ± 2.91	**<0**.**001**
Albumin (±SD)—g/L	42.04 ± 4.09	37.42 ± 2.49	34.33 ± 2.84	29.75 ± 5.51	**<0**.**001**
ALT (±SD)—mmol/L	95.76 ± 128.60	175.88 ± 587.19	192.99 ± 409.74	292.27 ± 497.06	0.087
TC (±SD)—mmol/L	4.53 ± 1.06	4.42 ± 0.91	3.98 ± 1.16	4.68 ± 1.15	0.542
TG (±SD)—mmol/L	1.69 ± 0.80	1.76 ± 1.27	1.51 ± 0.63	1.83 ± 2.04	0.546
LDL-C (±SD)—mmol/L	2.59 ± 0.87	2.67 ± 0.94	2.87 ± 1.36	2.61 ± 0.90	0.400
HDL-C (±SD)—mmol/L	1.02 ± 0.30	1.03 ± 0.28	1.07 ± 0.29	1.02 ± 0.22	0.640
Glucose (±SD)—mmol/L	7.44 ± 3.14	8.02 ± 3.02	8.79 ± 4.49	9.76 ± 6.66	**0**.**024**
Uric Acid (±SD)—μmol/L	348.05 ± 103.23	353.68 ± 128.47	354.96 ± 137.07	426.91 ± 213.08	**0**.**008**
SCr (±SD)—(μmol/L)	98.21 ± 83.44	113.12 ± 75.82	113.56 ± 69.87	167.39 ± 107.67	**<0**.**001**
HGB (±SD)—g/L	133.45 ± 16.87	127.21 ± 14.64	123.21 ± 16.79	118.62 ± 25.41	**<0**.**001**
WBC (±SD)—× 10^9^/L	11.64 ± 4.62	11.32 ± 4.98	11.82 ± 5.93	13.78 ± 7.35	0.067
PLT (±SD)—× 10^9^/L	205.73 ± 94.16	229.67 ± 108.44	190.52 ± 88.27	187.90 ± 103.51	0.061
LVEF (±SD)—%	41.96 ± 11.17	41.02 ± 10.85	39.11 ± 8.40	32.98 ± 12.10	**<0**.**001**
cTNI (±SD)—ng/mL	41.59 ± 33.41	36.14 ± 30.85	43.91 ± 28.92	48.12 ± 29.79	**0**.**016**
CRP (±SD)—mg/L	25.22 ± 25.69	34.88 ± 47.50	24.66 ± 27.47	42.54 ± 46.64	**0**.**021**
BNP (±SD)—pg/mL	600.69 ± 654.53	1,107.35 ± 1,307.80	1,184.35 ± 1,416.62	1,263.81 ± 1,788.19	**0**.**023**
Vasopressor used—no. (%)	66 (100.0)	66 (100.0)	66 (100.0)	66 (100.0)	1.000
Inotrope used—no. (%)	66 (100.0)	66 (100.0)	66 (100.0)	66 (100.0)	1.000
IABP or ECMO used—no. (%)	24 (36.4)	29 (43.9)	32 (48.5)	46 (69.7)	**0**.**001**
PCI success—no. (%)	46 (69.7)	46 (69.7)	44 (66.7)	32 (48.5)	**0**.**031**
IABP-SHOCK II score—no. (%)					**<0**.**001**
0–2	43 (65.2)	38 (57.6)	32 (48.5)	6 (9.1)	
3–4	17 (25.8)	21 (31.8)	19 (28.8)	31 (47.0)	
5–9	6 (9.1)	7 (10.6)	15 (22.7)	29 (43.9)	
MACE—no. (%)	36 (54.5)	35 (53.0)	41 (62.1)	52 (78.8)	**0**.**008**
Mortality—no. (%)	22 (33.3)	25 (37.9)	36 (54.5)	44 (66.7)	**<0**.**001**
Recurrent non-fatal myocardial infarction	4 (6.1)	2 (3.0)	1 (1.5)	2 (0.0)	0.162
Unplanned revascularization	4 (6.1)	2 (3.0)	1 (1.5)	2 (0.0)	0.162
Non-fatal stroke	6 (9.1)	7 (10.6)	8 (12.1)	6 (9.1)	0.929

RAR indicates red blood cell distribution width-to-albumin ratio; SBP, systolic blood pressure; DBP, diastolic blood pressure; RDW, red blood cell distribution width; ALT, alanine aminotransferase; TC, total cholesterol; TG, triglycerides; LDL-C, low-density lipoprotein cholesterol; HDL-C, high-density lipoprotein cholesterol; SCr, serum creatinine; HGB, hemoglobin; WBC, white blood cell count; PLT, blood platelet count; LVEF, left ventricular ejection fraction; MACE, major adverse cardiovascular events; cTNI, cardiac troponin I; CRP, C-reactive protein; BNP, B-type natriuretic peptide; IABP, intra-aortic balloon pump; ECMO, extracorporeal membrane oxygenation; PCI, percutaneous coronary intervention. Data are presented as the mean ± SD or *n* (%). *P* < 0.05 is marked in bold type.

### Relationship between RAR and in-hospital MACE and mortality in AMI-CS

Baseline characteristics of patients with and without MACE are shown in Table 2. Higher albumin levels and lower RDW/RAR were associated with lower incidence of MACE. Multivariate logistic regression (Table 3) demonstrated that elevated RAR was associated with increased MACE (Model 1: OR = 1.603, 95%CI 1.198–2.146, *P* = 0.002; Model 2: OR = 1.606, 95%CI 1.198–2.153, *P* = 0.002; Model 3: OR = 2.063, 95%CI 1.374–3.009, *P* < 0.001) and mortality (Model 1: OR = 1.882, 95%CI 1.414–2.507, *P* < 0.001; Model 2: OR = 1.978, 95%CI 1.473–2.657, *P* < 0.001; Model 3: OR = 2.033, 95%CI 1.341–3.084, *P* = 0.001). Compared with the lowest quartile, the highest RAR quartile conferred a 4.008-fold higher risk of MACE (95%CI 1.504–10.680, *P* = 0.006) and 3.354-fold higher risk of mortality (95%CI 1.414–7.958, *P* = 0.006) in Model 3.

**Table 2:** Baseline characteristics of the MACE and non-MACE AMI-CS patients.

Characteristics	Non-MACEs (*N* = 100)	MACEs (*N* = 164)	*P* value
Age (±SD)—years	64.68 ± 10.80	65.73 ± 13.30	0.486
Male sex—no. (%)	70 (70.0)	111 (67.7)	0.694
SBP (±SD)—mmHg	110.28 ± 19.28	109.32 ± 28.71	0.745
DBP (±SD)—mmHg	68.47 ± 13.87	67.48 ± 23.72	0.830
Hypertension—no. (%)	38 (38.0)	84 (51.2)	**0**.**037**
Diabetes mellitus—no. (%)	30 (30.0)	51 (31.1)	0.851
Current smoker—no. (%)	24 (24.0)	39 (23.8)	0.968
RAR (±SD)—%/g/dL	3.76 ± 0.81	4.17 ± 1.07	**<0**.**001**
RDW (±SD)—%	13.64 ± 1.76	14.10 ± 2.18	0.058
Albumin (±SD)—g/L	37.04 ± 4.40	35.18 ± 6.63	**0**.**013**
ALT (±SD)—mmol/L	148.80 ± 268.58	213.87 ± 521.58	0.183
TC (±SD)—mmol/L	4.03 ± 1.19	4.36 ± 0.99	**0**.**022**
TG (±SD)—mmol/L	1.66 ± 1.57	1.77 ± 0.68	0.480
LDL-C (±SD)—mmol/L	2.66 ± 0.93	2.70 ± 1.09	0.787
HDL-C (±SD)—mmol/L	1.04 ± 0.29	1.04 ± 0.27	0.926
Glucose (±SD)—mmol/L	9.07 ± 4.94	8.16 ± 4.40	0.124
Uric Acid (±SD)—μmol/L	365.65 ± 137.65	374.10 ± 163.09	0.665
SCr (±SD)—(μmol/L)	101.78 ± 62.09	136.05 ± 99.86	**0**.**002**
HGB (±SD)—g/L	125.66 ± 16.94	125.60 ± 21.03	0.982
WBC (±SD)—× 10^9^/L	9.64 ± 4.32	13.67 ± 6.16	**<0**.**001**
PLT (±SD)—× 10^9^/L	201.66 ± 94.07	204.46 ± 103.32	0.825
cTNI (±SD)—ng/mL	36.13 ± 28.57	46.29 ± 31.74	**0**.**009**
CRP (±SD)—mg/L	24.72 ± 34.64	43.48 ± 42.10	**<0**.**001**
BNP (±SD)—pg/mL	944.68 ± 1,091.00	1,096.59 ± 1,518.30	0.384
Vasopressor used—no. (%)	100 (100.0)	164 (100.0)	1.000
Inotrope used—no. (%)	100 (100.0)	164 (100.0)	1.000
IABP or ECMO used—no. (%)	40 (40.0)	91 (55.5)	**0**.**015**
PCI success—no. (%)	66 (66.0)	102 (62.2)	0.533
IABP-SHOCK II score—no. (%)			**<0**.**001**
0–2	68 (68.0)	51 (31.1)	
3–4	26 (26.0)	51 (31.1)	
5–9	6 (6.0)	62 (37.8)	
LVEF (±SD)—%	38.37 ± 8.08	39.47 ± 13.77	0.507

MACE indicates major adverse cardiovascular events; AMI-CS, acute myocardial infarction complicated with cardiogenic shock; SBP, systolic blood pressure; DBP, diastolic blood pressure; RAR, red blood cell distribution width-to-albumin ratio; RDW, red blood cell distribution width; ALT, alanine aminotransferase; TC, total cholesterol; TG, triglycerides; LDL-C, low-density lipoprotein cholesterol; HDL-C, high-density lipoprotein cholesterol; SCr, serum creatinine; HGB, hemoglobin; WBC, white blood cell count; PLT, blood platelet count; LVEF, left ventricular ejection fraction; cTNI, cardiac troponin I; CRP, C-reactive protein; BNP, B-type natriuretic peptide. Data are presented as the mean ± SD or *n* (%). *P* < 0.05 is marked in bold type.

**Table 3:** Multivariable logistic regression (95% CIs) for MACE and mortality according to the RAR index in AMI-CS patients.

Characteristics	Model 1		Model 2		Model 3	
	OR (95% CI)	*P* value	OR (95% CI)	*P* value	OR (95% CI)	*P* value
MACE						
RAR index	1.603 (1.198–2.146)	**0**.**002**	1.606 (1.198–2.153)	**0**.**002**	2.063 (1.374–3.009)	**<0**.**001**
RAR index (category)						
Q1	Ref		Ref		Ref	
Q2	0.941 (0.475–1.865)	0.861	0.910 (0.456–1.816)	0.789	0.903 (0.351–2.325)	0.833
Q3	1.409 (0.770–2.577)	0.266	1.417 (0.774–2.596)	0.259	1.014 (0.404–2.545)	0.976
Q4	2.846 (1.480–5.475)	**0**.**002**	2.910 (1.497–5.657)	**0**.**002**	4.008 (1.504–10.680)	**0**.**006**
*P* for trend		**0**.**011**		**0**.**008**		**0**.**009**
Mortality						
RAR index	1.882 (1.414–2.507)	**<0**.**001**	1.978 (1.473–2.657)	**<0**.**001**	2.033 (1.341–3.084)	**0**.**001**
RAR index (category)						
Q1	Ref		Ref		Ref	
Q2	1.220 (0.597–2.490)	0.586	1.075 (0.517–2.234)	0.847	0.920 (0.311–2.724)	0.880
Q3	2.173 (1.191–3.967)	**0**.**011**	2.259 (1.221–4.178)	**0**.**009**	1.595 (0.668–3.808)	0.2293
Q4	2.796 (1.555–5.030)	**0**.**001**	3.218 (1.746–5.931)	**<0**.**001**	3.354 (1.414–7.958)	**0**.**006**
*P* for trend		**<0**.**001**		**<0**.**001**		**0**.**039**

Model 1 (unadjusted), Model 2 (adjusted for age, sex and medical history), Model 3 (adjusted for age, sex, medical history, systolic/diastolic blood pressure, hemoglobin, white blood cell count, alanine aminotransferase, total cholesterol, triglycerides, serum creatinine, fasting blood glucose, cardiac troponin I, C-reactive protein, B-type natriuretic peptide). MACE indicates major adverse cardiovascular events; AMI-CS, acute myocardial infarction complicated with cardiogenic shock; RAR, red blood cell distribution width-to-albumin ratio; OR, odds ratio; CI, confidence interval. *P* < 0.05 is marked in bold type.

### Predictive value of RAR for MACE and mortality

**Figure 2:**
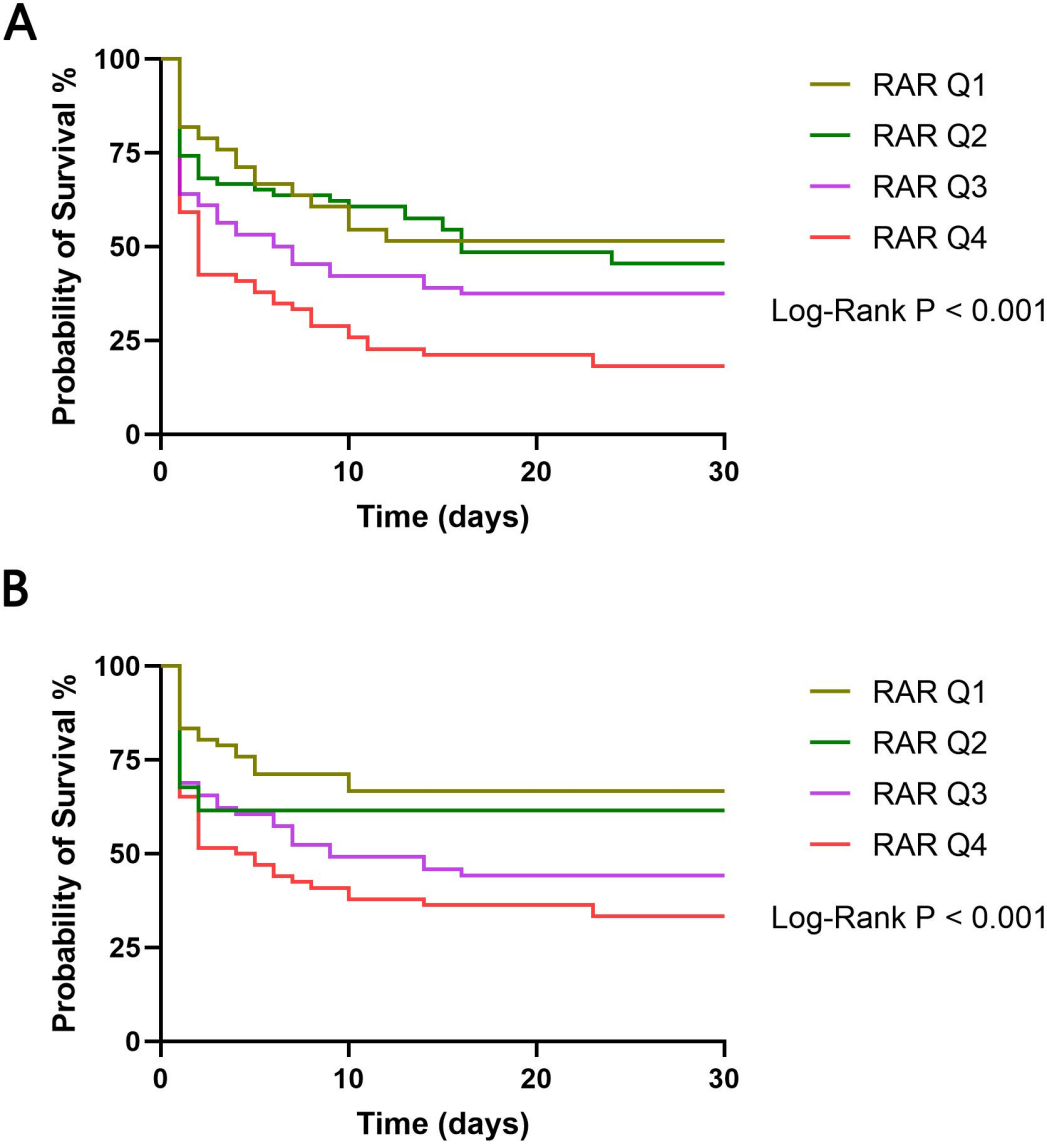
Kaplan–Meier survival curves for MACE and mortality during hospitalization across RAR quartiles. **(A)** Represents MACE; **(B)** represents mortality. RAR indicates red blood cell distribution width-to-albumin ratio; MACE, major adverse cardiovascular events.

**Figure 3:**
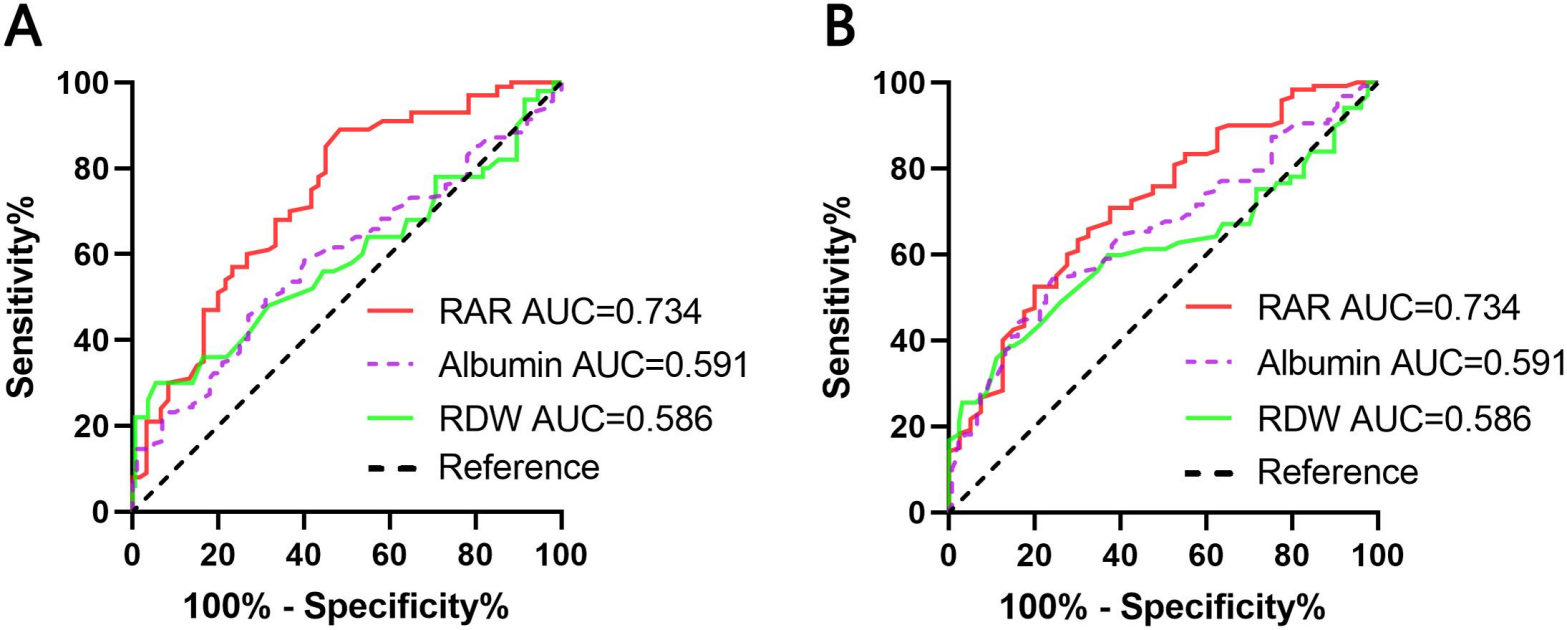
ROC curves for RAR predicting in-hospital MACE and mortalit. **(A)** ROC curves for MACE **(B)** ROC curves for mortality. RAR indicates red blood cell distribution width-to-albumin ratio; RDW, red blood cell distribution width; MACE, major adverse cardiovascular events.

Kaplan–Meier curves (Figure 2) showed that higher RAR quartiles were associated with higher in-hospital MACE and mortality (*P* < 0.001). ROC analysis was performed to assess predictive performance and determine optimal cutoffs. For MACE (Figure 3A), AUCs (95%CI) were 0.734 (0.653–0.815) for RAR, 0.586 (0.511–0.662) for RDW, and 0.591 (0.523–0.660) for albumin. The optimal RAR cutoff for MACE was 4.335 (sensitivity 89.0%, specificity 51.7%). For mortality (Figure 3B), AUCs were 0.716 (0.625–0.806) for RAR, 0.648 (0.559–0.738) for RDW, and 0.654 (0.578–0.721) for albumin. The optimal cutoff was 4.400 (sensitivity 83.3%, specificity 45.0%). RAR exhibited superior predictive ability compared with RDW and albumin (*P* < 0.05).

## Discussion

In contemporary emergency care, patients with suspected ACS are evaluated through an integrated approach including symptoms, ECG findings, and serial high-sensitivity troponin algorithms; therefore, hematologic indices such as RAR should be interpreted as prognostic adjuncts after AMI or AMI-CS identification rather than as diagnostic substitutes (24). This study demonstrated that elevated RAR was significantly associated with an increased risk of in-hospital MACE and mortality in patients with AMI-CS. Moreover, RAR exhibited superior predictive performance for adverse outcomes compared with RDW or albumin alone. These findings support the role of RAR as a convenient and reliable prognostic biomarker, and indicate it is a promising adjunctive candidate that requires prospective validation in this high-risk population.

RDW is a routinely measured hematological parameter that reflects erythrocyte size heterogeneity. Emerging evidence has linked elevated RDW to systemic inflammation, impaired microcirculation, nutritional disturbances, and poor cardiovascular prognosis (25, 26). Previous studies have reported that higher RDW levels predict increased mortality in patients with AMI (27, 28). However, evidence regarding the prognostic value of RDW specifically in AMI-CS remains limited. In the present study, we found that elevated RDW might be associated with worse outcomes in AMI-CS patients. Mechanistically, several plausible pathways may underlie this association. In AMI, acute coronary thrombosis results in severe myocardial ischemia-hypoxia injury, triggering intense inflammatory and oxidative stress that disrupts erythrocyte maturation. As the condition deteriorates into cardiogenic shock, reduced renal perfusion impairs renal function and disturbs erythropoietin synthesis, further suppressing erythropoiesis and elevating RDW (29). Additionally, increased RDW reflects diminished erythrocyte deformability and compromised microvascular perfusion, which exacerbate tissue hypoxia, disrupt the myocardial oxygen supply-demand balance, and increase the risk of adverse cardiovascular events (30). Furthermore, inflammatory cytokines including interleukin-1 (IL-1), IL-6, and tumor necrosis factor-α (TNF-α) are extensively released in AMI; these mediators inhibit effective hematopoiesis, aggravate erythrocyte maturation disorders, and promote anisocytosis (31, 32).

Albumin, another critical serum indicator, reflects nutritional status and systemic inflammation burden. Previous studies have confirmed that hypoalbuminemia is associated with adverse clinical outcomes in coronary artery disease and AMI (33, 34). Low albumin levels exert pro-inflammatory, pro-oxidative, and pro-thrombotic effects by enhancing platelet activation and aggregation, thereby increasing mortality risk in AMI (35). With clinical progression to cardiogenic shock, the deleterious impacts of hypoalbuminemia are further amplified. Concurrently, reduced oral intake and exaggerated systemic inflammation suppress albumin synthesis, establishing a vicious cycle that worsens patients' clinical conditions (36). Given the independent prognostic roles of RDW and albumin, their combined ratio, the RAR, may serve as a more comprehensive marker. In the present study, elevated RAR may reflect disease severity, systemic inflammation, malnutrition, renal dysfunction, hepatic congestion, hemodilution, or generalized systemic stress, which we hypothesized, may further exacerbate adverse in-hospital outcomes in patients with AMI-CS.

Consistent with prior clinical evidence, our study validated that elevated RDW and hypoalbuminemia are independently correlated with a higher risk of MACE among patients with AMI-CS. While both RDW and serum albumin served as independent prognostic biomarkers for adverse in-hospital outcomes, the combined RAR exhibited superior predictive performance. We further determined the optimal cut-off value of RAR (≥4.335) for predicting MACE and all-cause mortality in this critically ill cohort. Meanwhile, the IABP-SHOCK II score is the mainstay of risk stratification in AMI-CS patients in clinical practice. It includes age, creatinine, blood glucose, blood lactate, treatment variables, etc. However, calculating this score requires collection of multiple clinical parameters from patients. Our study found that patients in the highest RAR quartile also had significantly higher IABP-SHOCK II scores. As an easily accessible parameter calculated from routine peripheral blood laboratory results, RAR allows rapid and cost-free early risk stratification, and could assist in early prognostic assessment for AMI-CS patients. When interpreted alongside hemodynamic status, lactate levels, ECG, echocardiography findings, troponin dynamics, revascularization status, and established cardiogenic shock risk scores, RAR can help identify higher-risk patients who require intensified monitoring and aggressive interventions, including early revascularization, vasoactive support, and advanced circulatory support such as IABP or ECMO, to improve patient prognosis and reduce adverse events.

However, this study also has several limitations. First, as a two-center retrospective study, complete data for some variables were not available. Missing data on key variables related to AMI-CS severity and treatment, unit inconsistencies that required correction, heterogeneous treatment strategies, and uncertain outcome adjudication methods may all impact our results. Second, from a statistical perspective, the relatively modest sample size increases the risk of overfitting or multicollinearity in the adjusted model; in addition, if discharge status and variable length of stay were not properly accounted for, the validity of Kaplan–Meier analysis for in-hospital outcomes may be compromised, which could limit the generalizability of our findings. Third, some potential confounders, including selection bias and prior medication use, were not fully adjusted for in our analysis. Fourth, although our study confirmed that elevated RAR is independently associated with in-hospital MACE and mortality, the individual contributions of RDW and albumin to this prognostic value, as well as the underlying biological mechanisms, remain to be fully elucidated. Furthermore, serial dynamic measurements of RAR, combined with comparisons against other established prognostic biomarkers, would further improve the prognostic performance of RAR and shed more light on the underlying mechanisms. Future large-scale, multi-center, prospective studies are warranted to validate our findings and clarify the mechanistic basis of RAR in AMI-CS.

## Conclusion

In this two-center retrospective cohort of patients with AMI complicated by cardiogenic shock, higher admission RAR was associated with increased in-hospital MACE and mortality and showed higher discrimination than RDW or albumin alone. These findings suggest that RAR may be a simple candidate adjunctive prognostic marker in AMI-CS; however, prospective multicenter validation and assessment of incremental clinical utility are needed before routine implementation.

## Data Availability

The original contributions presented in the study are included in the article/Supplementary Material, further inquiries can be directed to the corresponding author/s.
